# Immunotherapy for Pediatric Brain and Spine Tumors: Current State and Future Directions

**DOI:** 10.1159/000528792

**Published:** 2022-12-22

**Authors:** Dagoberto Estevez-Ordonez, Sam E. Gary, Travis J. Atchley, Pedram D. Maleknia, Jordan A. George, Nicholas M.B. Laskay, Evan G. Gross, Rishi K. Devulapalli, James M. Johnston

**Affiliations:** ^a^Department of Neurosurgery, University of Alabama at Birmingham, Birmingham, Alabama, USA; ^b^Division of Pediatric Neurosurgery, Children's of Alabama, Birmingham, Alabama, USA; ^c^Heersink School of Medicine, University of Alabama at Birmingham, Birmingham, Alabama, USA

**Keywords:** Pediatric neuro-oncologist, Pediatric central nervous system tumors, Brain tumors, Immunotherapy, Immunovirothepary

## Abstract

**Background:**

Brain tumors are the most common solid tumors and the leading cause of cancer-related deaths in children. Incidence in the USA has been on the rise for the last 2 decades. While therapeutic advances in diagnosis and treatment have improved survival and quality of life in many children, prognosis remains poor and current treatments have significant long-term sequelae.

**Summary:**

There is a substantial need for the development of new therapeutic approaches, and since the introduction of immunotherapy by immune checkpoint inhibitors, there has been an exponential increase in clinical trials to adopt these and other immunotherapy approaches in children with brain tumors. In this review, we summarize the current immunotherapy landscape for various pediatric brain tumor types including choroid plexus tumors, embryonal tumors (medulloblastoma, AT/RT, PNETs), ependymoma, germ cell tumors, gliomas, glioneuronal and neuronal tumors, and mesenchymal tumors. We discuss the latest clinical trials and noteworthy preclinical studies to treat these pediatric brain tumors using checkpoint inhibitors, cellular therapies (CAR-T, NK, T cell), oncolytic virotherapy, radioimmunotherapy, tumor vaccines, immunomodulators, and other targeted therapies.

**Key Messages:**

The current landscape for immunotherapy in pediatric brain tumors is still emerging, but results in certain tumors have been promising. In the age of targeted therapy, genetic tumor profiling, and many ongoing clinical trials, immunotherapy will likely become an increasingly effective tool in the neuro-oncologist armamentarium.

## Introduction

Brain tumors in children are the most common solid tumors, accounting for up to 20% of childhood cancers, second only to leukemia [1, 2, 3, 4, 5, 6], represent the leading cause of cancer-related deaths in children [4, 5]. In the USA alone, the age-adjusted incidence rate of brain tumors in children is estimated at 6.14 cases per 100,000 and has steadily increased over time [1, 2, 3, 6].

Recent advances in therapy and diagnosis have led to improvements in survival for children with central nervous system (CNS) tumors [4]. In 2021, the fifth edition of the World Health Organization (WHO) Classification of Tumors of the Central Nervous System (CNS5) was published using a restructured classification with greater emphasis on molecular features [7]. This new edition better reflects current understanding of diagnostic categories based on important genetic features with significant relevance to prognosis and treatment.

While progress has been made for the management of children with CNS tumors, prognosis for many tumor types remains poor, and most standard treatment options have significant long-term negative effects [8, 9]. With the revolutionary development of immune-checkpoint inhibitors (ICI) establishing the era of immunotherapy for cancer treatment, many other approaches harnessing the immune system have been introduced and are at varying stages of development [10]. All of these developments have opened potential new approaches for the management of pediatric brain tumors. In this review, we highlight the current landscape of immunotherapy for the treatment of each specific type of pediatric brain tumor (Table 1).

## Choroid Plexus Tumors

Choroid plexus tumors (CPTs) are rare neoplasms arising from the choroid plexus epithelium within the ventricles. CPTs have an incidence of 0.3 per million and comprise only 0.5% of all intracranial tumors [11, 12]. However, in the first year of life, CPTs represent 12–20% of brain tumors with median diagnosis age of 3.5 years and a male:female ratio of 1.2:1 [13, 14, 15]. Patients with CPTs typically present with symptoms of hydrocephalus such as headache, ataxia, and diplopia secondary to cerebrospinal fluid (CSF) overproduction or CSF outflow obstruction [16]. The 2021 WHO classification of CPTs remains largely unchanged from prior years and is based on histological findings: choroid plexus papilloma (CPP, WHO grade I), atypical CPP (aCPP, WHO grade II), and choroid plexus carcinoma (CPC, WHO grade III) [7]. The one-, five-, and 10-year projected survival rates for CPP patients are 90, 81, 77%, respectively, compared to 71, 41, 35% for CPC patients [15]. For patients with CPP, a primary gross total resection alone is often curative. Conversely, patients with CPC are typically treated with resection followed by chemotherapy and/or radiotherapy, both of which have been shown to improve overall survival compared to surgical resection alone [15, 17]. However, CPC patients undergoing radiation and chemotherapy (various multiagent protocols including agents such as cyclophosphamide, cisplatinum, carboplatinum, etoposide, lomustine, and vincristine among others) following incomplete resection had a 2-year overall survival rate of only 63% [17].

Although the literature is sparse, there are currently four clinical trials investigating immunotherapies against CPTs. Of these clinical trials, there is one phase 2 study evaluating the efficacy of ICI nivolumab, a monoclonal antibody against the programmed death-1 (PD-1) receptor expressed on the surface of activated human lymphocytes (NCT03173950). Chimeric antigen receptor (CAR)-T-cells that are engineered to target tumor-specific neoantigens, independent of major histone compatibility (MHC) presentation of neoantigens, is also a promising avenue for brain tumor treatment. There are three ongoing phase I clinical trials, all testing CAR-T-cell therapies delivered via an indwelling catheter into either the tumor resection cavity or ventricular system in both pediatric and young adult patients. These are EGFR806-specific (NCT03638167), B7H3-specific (NCT04185038), and HER2-specific (NCT03500991) CAR CD4+ and CD8+ T cells.

Beyond clinical trials, there are several noteworthy investigational approaches with translational potential in mouse models of CPTs. Schell et al. [18] reveal a promising immunotherapy against CPTs using adoptive T-cell transfer (ADT). In transgenic mice expressing simian virus 40 (SV40) oncogene, whole-body irradiation (WBI) followed by intravenous injection with T Ag-specific donor CD8+ T cells resulted in rapid, high-level T cell accumulation within the brain, CPC tumor elimination, persistence of T cells at tumor sites, and prevention of tumor recurrence [18, 19]. Cozza et al. [20] further showed intraperitoneal injection of anti-CD40 igG prior to CD8+ T cell injection − ADT − achieved similar levels of T-cell accumulation and CPT elimination while significantly extending survival in absence of WBI. However, ADT following WBI promoted high-level T cell accumulation and long-term protection from tumor recurrence [20].

## Embryonal Tumors

### Medulloblastoma

Medulloblastomas (MBs) are the most common malignant pediatric brain neoplasm, accounting for as many as 10–20% of brain tumors [21, 22, 23, 24]. These tumors originate from the cerebellar vermis and often invade the 4th ventricle resulting in obstructive hydrocephalus and numerous other neurologic deficits including ataxia, dysmetria, and cranial neuropathies [25]. MBs may metastasize through the craniospinal axis [21]. The conventional treatment of MB includes surgical resection with adjuvant chemoradiation, resulting in a 5-year survival rate of ∼70–75%; however, survival rates are molecular subgroup specific ranging from ∼50 to 95%. The WHO previously classified MB into four histological groups: large cell, anaplastic, nodular desmoplastic, and extensive nodularity [21, 26, 27]. The WHO classification has more recently expanded to incorporate molecularly defined subtypes: WNT-activated, sonic hedgehog (SHH) activated, and group 3 and group 4 referred to as non-WNT/non-SHH [7, 26]. SHH-activated is further subdivided into TP53-wildtype and TP53-mutant [7].

The different molecular characteristics of MB are also related to varying embryologic origins. WNT-activated tumors arise from the dorsal brainstem [28]. SHH-activated tumors originate from granule neuron precursor cells (GNPCs) from the cerebellum [28]. Group 3 tumors arise from GNPCs but via a non-SHH pathway, and portend a poor prognosis with an approximate survival rate of 50% [28]. These tumors express genes resembling retinal rod precursor cells [29]. Group 4 MBs originate from GNPCs but their gene expression resembles cerebellar glutamatergic granule neurons in late fetal development [29]. WNT tumors are the least common but harbor the best prognosis with 95% 5-year survival. Group 4 is the most common subtype [21].

The treatment paradigm for MB is complex, involving risk stratification for oncological management protocols, and has continually evolved over the past few decades, but immunotherapy remains investigational for affected patients. Historically, following maximal safe resection, children over 3 years received external beam radiation to the craniospinal axis with a multidrug chemotherapy regimen which usually includes cisplatin, vincristine, cyclophosphamide, and lomustine [21, 30, 31]. Patients with high-risk disease were given larger boosts of craniospinal radiation [21, 30, 31]. Children under 3 years receive surgery and chemotherapy without radiation. However, there has been increasing focus on radiation-sparing regimens for children with MB regardless of age. Children with SHH-activated MB may be effectively treated with adjuvant chemotherapy alone. While group 3 and 4 MBs may still require aggressive postsurgical therapies [32, 33]. Treatment is associated with significant cognitive, neuroendocrine, and neurosensory abnormalities and secondary malignancies. Despite these regimens, recurrent and refractory disease presents a major problem, and ∼30% of patients succumb to their disease [34].

MBs classically have a highly heterogeneous genetic landscape and are often immunosuppressive in nature, making the development of targeted immunotherapy challenging. Nevertheless, various pathways have demonstrated promise. Though ICIs have demonstrated promise in other pediatric brain tumors, MBs classically express less PD-L1 than other CNS neoplasms [21, 35]. Though some studies have found PD-1 blockade may be possible in SHH-activated MB, ICIs have not demonstrated substantial efficacy in treatment of pediatric MB (29) While cytotoxic T cells can infiltrate MB, their activation has not been correlated with increased survival [21, 36]. Regardless, ongoing trials of MB patients are underway investigating nivolumab and durvalumab (NCT03173950 and NCT02793466). One phase I trial (NCT02359565) is investigating pembrolizumab in pediatric patients with recurrent or refractory MB. Treatment with nivolumab with and without ipilimumab targeting cytotoxic T-lymphocyte antigen 4 is actively being studied (NCT03130959) in patients with high-grade CNS malignancies, including MB [37]. A phase I study investigating indoleamine 2,3-dioxygenase, an ICI, in combination with radiotherapy for pediatric brain tumors was recently expanded to a phase II trial in August 2019 (NCT04049669).

SHH-activated MB is associated with higher infiltration of T cells, macrophages, and fibroblasts compared to other subtypes [37]. Additionally, greater expression of inflammation-related genes (CD14, PTX3, CD4, CD163, CSF1R, and TGFB2) is observed in tumors of the SHH subgroup [37]. Natural killer (NK) cells may prove an adequate target for immunotherapy in MB. These tumors demonstrate downregulation of MHC-I that may make malignant cells more susceptible to NK-mediated activity. A recently completed phase I trial (NCT02271711) investigated autologous NK cells delivered via 4th ventricular catheter following surgery for recurrent MB. The investigators found no dose-limiting toxicities, but almost all patients demonstrated progressive disease despite therapy [38].

CAR-T therapy is also being investigated for the treatment of MBs. Notably, CAR-T-cell therapy does not necessitate a robust systemic immune response and may be more suitable for tumors that lack high mutational burden, like MB [39]. CAR-T-cells have demonstrated promise against high-grade gliomas (HGGs) in targeting HER2 [40]. This receptor is often overexpressed in certain MB subtypes and may be a potential target [40]. One study showed efficacy of CAR-T-cells engineered for HER2 in murine models with MB without significant neurotoxicity [41]. An ongoing trial (NCT03500991) is evaluating HER2-specific CAR-T therapy in HER2-positive recurrent CNS tumors including MB. EGFR-positive CNS tumors are also under investigation for potential treatment with CAR-T-cells (NCT03638167). Challenges remain in developing CAR-T-cell therapies that can infiltrate the specialized tumor microenvironment of MB [42]. Other shortcomings of CAR-T therapies include antigenic escape and neurotoxicity [39]. Dendritic cell (DC) vaccines and autologous T cell transfers are currently also being studied in a phase I clinical trial for patients with recurrent MB. In one study, patients with recurrent MB are being treated with autologous tumor-specific T cell immunotherapy (TTRNA-xALT) and concomitant total tumor RNA-loaded DC vaccine to boost the immune response (NCT01326104). Other ongoing vaccine trials include Cytomegalovirus (CMV) RNA-Pulsed DCs with tetanus toxoid preconditioning and granulocyte macrophage-colony stimulating factor (GM-CSF) (NCT03615404).

Studies have also investigated radiolabeled antibodies as a therapeutic option. Two phase II trials (NCT00058370; NCT00445965) have investigated the utility of intrathecal radioimmunotherapy (IR) using radiolabeled monoclonal antibodies to target tumor cells and deliver local radiation in patients who have previously undergone surgery for MB. Results from these studies show favorable dosimetry to CSF as well as clinical therapeutic potential. Following a phase II study investigating intraventricular infusion of ^131^I-3F8 for MB, the authors found decreased risk of death in patients in radiographic remission compared to those with radiographic disease [43].

Oncolytic viruses (OVs) are also under study as novel therapeutic agents against MB. In addition to direct oncolytic effects from selective infection of tumor cells, viruses may have the added benefit of overcoming tumor-mediated immunosuppression, promoting infiltration of tumor infiltrating lymphocytes (TIL) and converting from a “cold” to “hot” tumor microenvironment. Poliovirus's affinity for the CD155 receptor in MB has demonstrated tumor growth inhibition by rhinorvirus recombinant variant of polio (PVSRIPO) in (NCT03043391) [44]. Poliovirus receptor concentration is highest in the WNT and group 3 subtypes suggesting these groups as potential targets [21]. A reengineered reovirus, reolysin, is being studied in patients with high-grade CNS malignancies, including MB (NCT02444546). Based on encouraging results seen in supratentorial HGG, a phase I trial of G207 HSV1 administered via intratumoral catheter infusion in children with recurrent high-grade cerebellar tumors is underway (NCT03911388). A modified measles virus is also being investigated in a phase I trial but the results are still pending (NCT02962167). Orthotopic murine models of SHH-subtype MB treated with intratumoral myxoma virus or with dsRNA reovirus have demonstrated prolonged survival [37]. Other OVs including rodent parvovirus H-1 (H-1PV) and adenovirus Ad5 delta-24 have demonstrated in vitro lytic effects against multiple MB subtypes [45].

### Atypical Teratoid/Rhabdoid Tumor

Classified as a grade IV primary CNS tumor, atypical teratoid/rhabdoid tumors (AT/RT) are very rare with an incidence of 0.5 per million followed by poor prognosis at a survival rate of less than 1 year [46]. These tumors arise from embryonic tissue including mesenchymal, neuroectodermal, and epithelial precursors. Histologically, these are characterized by the presence of a “rhabdoid” component, referring to groups of epithelioid cells with eosinophilic inclusions. Immune markers typically include vimentin, epithelial membrane antigen, and smooth muscle actin [46].

AT/RTs have shown an abundance of CD8+ T cells and eosinophil infiltration which further suggests immunotherapy as a potentially effective approach to targeting this aggressive tumor [47]. Autologous DC vaccines, created with tumor cells cultured in interleukin 4 (IL4), granulocyte-macrophage colony-stimulating factor preceding maturation with IL-1B and tumor necrosis factor alpha, were shown to prolong survival and remission control in three young children with safe administration an minimal side effects [48]. B7-H3/CD276 is highly expressed in AT/RT cells and targeting this protein via CAR-T cells has shown antitumoral effects in vitro and in xenograft mice models. The study further suggests intracerebroventricular over intravenous delivery to increase efficacy and reduce systemic cytokine effects of CAR-T based therapies [49]. ICIs, such as PD-1 blockade, have also shown a reduction in tumor growth in mice models [50]. Oncolytic measles virus has shown antitumoral effects against AT/RTs in xenograft mice models and is currently being studied in a phase I clinical trial (NCT02962167) [51]. Oncolytic herpes virus rRp450 has shown antitumoral effects with prolonged survival and increased efficacy of cyclophosphamide in xenograft models [52]. Other clinical trials are currently assessing efficacy of nivolumab (anti-PD-1; NCT02834013, and NCT02834013), ipilimumab (anti-CTLA-4; NCT02834013), tiragolumab (anti-TIGIT; NCT05286801), atezolizumab (anti-PD-L1; NCT05286801), and lenalidomide (immune modulator; NCT00100880) for the treatment of AT/RT.

### Primitive Neuroectodermal Tumors

Primitive neuroectodermal tumors (PNETs) are a rare group of grade IV malignant tumors that arise from primitive neural cells as a remnant from gestation [53]. Examples of tumors that are classified as PNETs include medulloepithelioma, CNS neuroblastoma with FOXR2 activation, and other embryonal tumors. Due to the rare nature of these tumors, much of current treatment approaches have been inferred from the experience with MB and include a combination of surgery followed by chemoradiation [54, 55].

Targeting PD-1/PD-L1 has been shown to result in efficient antitumor immunity in neuroblastoma cells [56, 57]. Other antibodies have also been developed and tested for the treatment of high-risk neuroblastoma (HRNB) targeting GD2, a surface antigen expressed on tumors of neuroectodermal origin [58, 59]. Additionally, cytokines such as GM-CSF and interleukin 2 (IL2) have been shown to further enhance this antibody mediated cytotoxicity when targeting GD2 [60]. Yu et al. [61] further showed the improved event-free and overall survival in children with HRNB receiving this combination therapy which eventually led to the FDA approval of anti-GD2 antibody and cytokine immunotherapies such as dinutuximab and naxitamab-gqgk for the treatment of HRNB [61, 62]. However, these trials had few patients with metastatic disease to the brain and less is known about their effect on primary CNS neuroblastoma and has not lead to changes in treatment approaches.

There are numerous other immunotherapies currently under investigation in clinical trials. DC vaccines and autologous T cell transfers are currently under a phase II clinical trial for patients with recurrence of PNET following radiation (NCT01326104) [21]. Durvalumab is an FDA approved PD-L1 inhibitor for other tumor types and is currently under clinical trial for PNETs (NCT02793466). CAR-T therapy targeting HER2 (NCT03500991) and EGFR806 (NCT03638167), overexpressed pathways regulating cell growth in tumors, are under a phase I trial. Radioimmunotherapy has been of focus in a few clinical trials with iodine monoclonal antibody ^131^I-3F8 or dosimetry-guided octreotide peptide receptor (NCT00445965). Specific to CNS neuroblastoma patients, ^131^I-3F8 and iodine monoclonal antibody ^131^I-8H9 have shown improved overall survival up to a median of 33 months following CNS neuroblastoma relapse which is greater than the 6-month median survival of the control group [63].

## Cranial Nerve Sheath Tumors

Nerve sheath tumors arise from myelin-producing Schwann cells and are benign in 85–90% of clinically symptomatic cases [64]. They have an overall incidence of 10.9 per million with peak incidence of 29.3 per million in the 65- to 74-year age range and no difference in incidence between sexes [65]. Nerve sheath tumors are subdivided into two major categories, schwannomas and neurofibromas, which are distinct in their histopathological growth. Schwannomas typically grow in an eccentric fashion without nerve fiber involvement while neurofibromas histologically show an intermingling of nerve fibers, fibroblasts, mast cells, and perineurial-like cells [66, 67]. Benign schwannomas comprise 89% of all nerve sheath tumors with surgical resection or stereotactic radiosurgery showing excellent outcomes [68, 69, 70]. However, prognosis is considerably worse in patients with cancer-predisposition syndromes such as neurofibromatosis (NF) 1 or 2 and schwannomatosis.

Multiple immunotherapies have been used off-label for treatment of nerve sheath tumors. Bevacizumab, a monoclonal antibody against vascular endothelial growth factor (VEGF) with antiangiogenic properties, has been implicated as an antitumor immune modulator in patients with ovarian cancer, colorectal cancer, and other malignant effusions [71, 72, 73]. Angiogenesis is critical for tumor growth, and microvessel density as well as vascular permeability have been identified as driving factors for vestibular schwannoma growth [74, 75]. A meta-analysis of eight studies using bevacizumab to treat NF2 reported tumor partial regression in 41% of patients, stable tumor size in 47%, and tumor progression in only 7%. From audiometric data, they report hearing improvement in 20%, stability in 69%, and additional hearing loss in only 6%, supporting bevacizumab's efficacy in treating NF2 schwannomas [76]. Bevacizumab has been tested as a vaccine injected subcutaneously to treat schwannomas in NF2 patients, demonstrating safety but only limited effects on schwannoma growth [77].

There are numerous phase I and II clinical trials testing immunotherapies against nerve sheath tumors. The phase II trials, all of which are currently recruiting, include the anti-CD40 agonist mAb sotigalimab (APX005M) with doxorubicin (NCT03719430), combination immunotherapy using anti-CTLA4 mAb ipilimumab with anti-PD-1 mAb nivolumab (NCT02834013), and antigen-specific T cells CAR-T/CTL with DCvac (NCT04085159). There is one active phase I trial testing anti-VEGF mAb bevacizumab with temsirolimus alone or in combination with valproic acid or cetuximab (NCT01552434). The remaining phase I trials, all currently recruiting, include B7H3-specific CAR-T cells alone or in combination with CD19-specific CAR-T cells (NCT04483778), EGFR-specific CAR-T cells alone or in combination with CD19-specific CAR-T cells (NCT03618381), combination immunotherapy using anti-CTLA4 mAb ipilimumab and anti-PD-1 mAb nivolumab (NCT04465643), anti-CTLA4 mAb ipilimumab with BO-112 prior to surgical resection or radiotherapy (NCT04420975), and B7H3-specific CAR-T cells after lymphodepleting chemotherapy (NCT04897321).

Preclinical trials have also demonstrated promise for the use of immunotherapeutics in treatment of nerve sheath tumors. Ahmed et al. [78] showed that intratumoral injection of murine schwannomas with the live attenuated Salmonella typhimurium strain VNP20009 led to increased tumor infiltration of CD45+ and CD68+ leukocytes as well as decreased tumor angiogenesis, tumor growth, and increased tumor cell apoptosis. They found similar therapeutic effects on distal, uninjected tumors which they suggest is induction of antitumor memory immune response. Furthermore, they report an additive therapeutic effect of intratumoral *S. typhimurium* injection in combination with intraperitoneal injection of anti-PD-1 monoclonal antibody, suggesting efficacy of combination immunotherapeutics. Immunohistochemical analysis on resected VSs has also provided insights on potential future VS immunotherapies. Phenotypically aggressive schwannomas show significant association between percent PD-L1 positivity, tumor progression, and unfavorable House-Brackmann facial nerve function highlighting the PD-1/PD-L1 axis as a promising target for future immunotherapeutics [79]. Others have found that VSs with strong expression of the immunoglobulin-like immune suppressive molecule B7-H1 are less responsive to stereotactic radiation, suggesting a role of B7-H1 in tumor resilience and immune tolerance [80].

## Ependymal Tumors

Ependymomas are derived from ependymal cells and are the third most common pediatric brain tumor [81]. These tumors typically arise in the ventricles but can also arise within brain parenchyma [82]. Due to their propensity to arise from different areas of the CNS, their clinical presentation varies widely. Based on genetic mutations and anatomic variations, ependymomas are classified into posterior fossa, supratentorial, and spinal ependymomas. Current WHO classification for ependymal includes supratentorial ependymoma (ZFTA fusion-positive), supratentorial ependymoma (YAP1 fusion-positive), posterior fossa ependymoma (group PFA), posterior fossa ependymoma (group PFB), spinal ependymoma (MYCN-amplified), myxopapillary ependymoma, and subependymoma [7].

Current treatment of ependymal involves surgical resection with adjuvant radiation therapy. Subependymal tumors have an excellent prognosis even in the absence of treatment; however, supratentorial ependymomas tend to have a poorer prognosis despite surgery and adjuvant radiation therapy. Effective targeted therapy has proven difficult in ependymomas, as there is significant molecular heterogeneity within this class of tumor. One area of promise is that of cancer vaccinations. A phase I study of patients with recurrent ependymoma receiving vaccination with Human Leukocyte Antigen A2 restricted peptides combined with imiquimod is currently underway (NCT01795313). In this study, a peptide-based immunotherapy targeted against cytotoxic T lymphocyte (CTL) epitopes of three tumor-associated antigens was created. The first two targets, IL-13Rα2 and EphA2, are tumor-associated antigens that are overexpressed in malignant tumors but not normal brain tissue [83]. Survivin, which is an apoptosis inhibitory protein expressed in many types of cancers, was used as the third target. Based on immunohistochemical staining of 19 pediatric ependymomas, 84% overexpressed IL-13Rα2, 95% overexpressed EphA2, and 95% overexpressed survivin [84]. Another study using vaccination with tumor lysate pulsed DCs showed significant promise, as 75% of children with recurrent ependymoma survived greater than 18 months [85].

Locoregional CSF delivery of CAR-T cell therapy has been evaluated in a mouse model with posterior fossa ependymoma. This proved effective, with all mice exhibiting tumor regression at 1 month and up to 40% of mice becoming tumor-free at endpoint [86]. Based on this research, a phase I trial evaluating the efficacy of HER2-specific CAR-T cells for children with ependymoma is currently recruiting participants (NCT04903080). A phase I clinical trial that will assess the efficacy of HER2-specific CAR-T cell locoregional immunotherapy in patients with HER2-positive recurrent or refractory pediatric CNS tumors, including ependymomas, is also currently in the recruitment phase (NCT03500991).

Oncolytic immunovirotherapy has also been assessed in these tumors. A phase I trial was conducted on pediatric patients with malignant glioma and recurrent ependymoma using aglatimagene besadenovec (AdV-tk) (NCT00634231) [87]. Gene-mediated cytotoxic immunotherapy via local delivery of AdV-tk induces a T cell dependent tumor immunity. AdV-tk is an adenoviral vector that expresses the herpes simplex virus thymidine kinase gene [87]. This approach shows promise in the treatment of ependymoma, especially when combined with surgical debulking and radiation.

Among other approaches include a pilot study on stimulation of immune cells with GM-CSF on ependymoma (NCT04408092) and autologous expansion of NK cells through a phase I trial (NCT02271711). Nine patients were enrolled in this trial, and they received treatment 3 times per week. All patients showed an increase in size of the lesion at the end of therapy [38].

## Germ Cell Tumors

Intracranial germ cell tumors (iGCT) comprise about 1% of primary pediatric brain tumors and are commonly located in the pineal gland and suprasellar region [88]. iGCTs are classified as either germinoma or nongerminomatous germ cell tumors (NGGCT), with germinomas making up between 50 and 60% of all iGCT. NGGCTs are further classified as embryonal carcinoma, yolk sac tumor, choriocarcinoma, teratoma (mature and immature), and mixed GCT. Prognosis varies widely within this group of tumors, with germinomas having a 90% survival rate and NGGCTs having between a 40–70% survival rate [89]. Treatment strategy also varies greatly between these two groups of tumors, as germinomas are regularly cured with radiation therapy alone while only 20–40% of NGGCTs are cured with radiation [90].

Immunotherapy has been explored as a novel approach to treating intracranial GCTs [91]. CD30+ is a member of the tumor necrosis factor receptor superfamily and is a defining marker of embryonal carcinoma. Brentuximab-vedotin, a novel antibody-drug conjugate, was successfully used to treat a patient with Down's syndrome and CD30+ embryonal carcinoma [92]. Palbociclib was successfully administered to a patient with growing teratoma syndrome [93, 94]. DC-based immunotherapy was performed on a patient with a previous histological diagnosis of germinoma and recurrence of malignant GCT. This therapy resulted in progressive decrease in tumor size and rapid decrease in tumor size and decreased serum beta-hCG from 232.2 ng/mL on admission to around 50 ng/mL 30 days after treatment [95]. Intracranial germinomas exhibit a high level of TIL [96]. Immunohistochemical staining of intracranial germinomas revealed that 90% stained positively for programmed death receptor ligand 1 (PD-L1), indicating that anti-PD-1 and anti-PD-L1 may have a future role in the treatment of intracranial germinomas [97]. A phase I clinical trial that will assess the efficacy of HER-2-specific CAR-T Cell locoregional immunotherapy in patients with HER2-positive recurrent or refractory pediatric CNS tumors (NCT03500991), including germ cell tumors, is currently in the recruitment phase.

## Gliomas

### Circumscribed Astrocytic Gliomas

Circumscribed astrocytic gliomas constitute low-grade tumors with overall favorable prognosis. According to the 2021 WHO classification, these include Juvenile Pilocytic Astrocytic (JPA), subependymal giant cell astrocytoma, and pleomorphic xanthoastrocytoma [7]. Of these, JPAs are the most common in pediatric patients and most often found in the posterior fossa tumors [98]. The main treatment approach is complete surgical resection, when possible, with subsequent observation. When complete resection without risk of neurological injury is not possible, then resection of the nodule alone without the cyst wall is an acceptable alternative approach [99]. Risk of recurrence is low for these tumors; however, in such instances, subsequent surgical resection is often preferred. Given its long-term sequalae, adjuvant radiation is avoided unless there is evidence of malignant progression [98]. JPA has an association with Neurofibromatosis 1 (NF1), with afflicted patients being susceptible to other tumors including low-grade gliomas (LGG) in the optic nerve, commonly referred to as optic pathway gliomas (OPG). Given the benign nature of JPAs, ability to often achieve adequate resection, and low recurrence rates other treatment avenues have mostly been explored in rare instances of recurrent or metastatic JPA [100]. A phase II clinical trial (NCT02343224) testing the efficacy of pegylated interferon in children with recurrent or refractory and progressive JPA and OPG was completed recently in January 2022. Primary results were presented at the Society of Neuro-Oncology meeting [101]. Pegylated interferon is a modification to interferon alpha or beta molecule where polyethylene glycol is added, which lengthens the duration of activity following administration [102].

### Pediatric-Type Diffuse LGG (Including OPG)

LGGs in pediatric patients include diffuse astrocytoma (MYB- or MYBL1-altered), angiocentric glioma, polymorphous low-grade neuroepithelial tumor of the young, and diffuse low-grade glioma, MAPK pathway-altered [7]. They comprise up to a third of pediatric brain tumors [103]. In general, prognosis for these tumors is favorable with 10-year survival between 85 and 96% [1]. However, as a result of disease progression or treatment complications, children often experience significant long-term negative affects to their quality of life [104]. Therefore, this is an area in dire need of improvement in our current approach to treatment. When feasible, maximal safe surgical resection is preferred as first line treatment. However, many of these involve eloquent areas in children, including the optic pathways and deep midline structures not amenable to surgical resection [105]. Radiation has been a mainstay treatment for LGG, despite its long-term negative sequelae [105]. Chemotherapy is also frequently used to treat LGG with common regimens including carboplatin and vincristine, a combination of thioguanine, procarbazine, CCNU, and vincristine or vinblastine alone [105]. Many of the ongoing efforts in immunotherapy have significant overlap with efforts on the treatment of pediatric-type diffuse HGGs; therefore, most of the discussion on immunotherapy is detailed in the HGG section of this review. The remainder of this section will focus on recent advances on OPGs, a subtype of LGG.

There is a significant association of OPGs with NF1 [106]. OPGs are the most frequent CNS tumor in children with NF1, with up to 20% of NF1 patients afflicted by an average age of 5 years [107]. Clinical manifestations and challenges in treatment are related to the direct or indirect involvement of several eloquent structures such as the optic pathways and hypothalamus. This severely limits the role of surgical treatment and standard radiation therapy. Therefore, chemotherapy and low-dose radiation are often used only after documented progression or progressive neurological decline. Radiation is also avoided in younger children due to its long-term sequelae. The first-line chemotherapy is usually a combination of carboplatin and vincristine [106]. There are many ongoing clinical trials, several of which are currently focused on targeting the MAPK pathway due to the alterations frequently encountered in these tumors [107]. Immunotherapy approaches have included the use of antibodies such bevacizumab in combination with concurrent vinblastine in an ongoing phase II clinical trial (NCT02840409). A phase II clinical trial (NCT02343224) testing the efficacy of pegylated interferon completed recently in January 2022. Preliminary results are inconclusive and median survival has not been reached yet. However, the authors report that no complete responses or partial responses were seen and 2 out of 9 patients had prolonged stable disease up to 75 months [101]. Other exploratory approaches include polyinosinic-polycytidylic acid stabilized with carboxymethylcellulose and poly-L-lysine (poly-ICLC) [108, 109]. Poly-ICLC is a stable double-stranded RNA molecule as a result of its polyinosinic acid homopolymer annealed to a polycytidylic acid homopolymer [108]. This molecule binds to endosomal Toll-like receptor (TLR)-3 and the cytoplasmic receptors retinoic acid-inducible gene I (RIG-I), thus mimicking viral infection and inducing a potent inflammatory response with secretion of type I interferon (IFN) and several other pro-inflammatory cytokines [110]. A phase II trial of poly-ICLC in children with recurrent LGG, including OPGs, recently finished enrollment (NCT01188096), and preliminary results are encouraging with final results will likely to be available soon [101].

### Pediatric-Type Diffuse HGG

Pediatric-type diffuse HGGs represent the most aggressive and lethal type of brain tumors in children. Pediatric diffuse HGG (pHGG) has recently been shown to contain distinct molecular features compared to adult HGG and therefore potentially different treatment susceptibilities [111, 112]. The fifth edition of the WHO Classification of Tumors of the CNS, therefore, separated pHGG into four distinct categories: diffuse midline glioma (DMG) histone 3 (H3) lysine 27 (K27)-altered, diffuse pediatric-type HGG (H3-wildtype and Isocitrate dehydrogenase [IDH]-wildtype), diffuse hemispheric glioma H3 glycine 34 (G34)-mutant, and infant-type hemispheric glioma [7]. Outcomes for pHGG have not improved for over 30 years, indicating the need for targeted therapies that achieve durable responses [113]. Below, we review immunotherapy approaches for DMG and H3 wildtype and IDH wildtype HGG, the most common and intensively researched forms of pHGG.

### DMG H3 K27 Altered

DMG typically presents in children at a median age of diagnosis of 6–7 years [114]. DMG is the most rapidly fatal primary brain tumor in children, with a median overall survival of 11 months and a 5-year overall survival of just 1–2% [112, 115, 116]. Up to 80% of DMG tumors arise in the pons and are subclassified as diffuse intrinsic pontine glioma (DIPG) [116]. Because of its anatomical proximity to the brainstem, surgery is contraindicated, and radiation and chemotherapy have shown no significant overall survival benefit [117].

### Adoptive Cell Transfer

Recent preclinical and phase I trial findings suggest a promising method for treatment of DIPG is ADT, specifically transfer of CAR-T cells that are engineered to target DMG-specific neoantigens independent of MHC presentation of neoantigens. Disialoganglioside 2 (GD2) was identified as a highly expressed neoantigen in four different patient-derived cell cultures of H3K27 altered DIPG, and treatment of orthotopic mouse DIPG xenografts with anti-GD2 CAR-T cells demonstrated nearly double (50 vs. 30 days) median overall survival compared to treatment controls before ending the treatment duration due to development of graft versus host disease in the anti-GD2 CAR-T cells treatment group [118]. A first-in-human phase I clinical trial (NCT04196413) of intravenously administered anti-GD2 CAR-T-cells (1 × 106 CAR-T cells/kg) demonstrated safety and efficacy with side effects manageable in the inpatient setting and clinical and radiographic improvement in 3 out of 4 patients with H3K27 altered DIPG or spinal cord DMG [119]. Side effects included previously published inflammatory syndromes caused by CAR-T cell therapy, most notably tumor inflammation-associated neurotoxicity, which was managed with corticosteroids and monoclonal antibodies directed against IL-1 (anakinra) and IL-6 (tocilizumab or siltuximab). Importantly, off-tumor side effects were not observed, indicating a high degree of tumor specificity of the anti-GD2 CAR-T cells likely due to the requirement of high expression of GD2, which was unique to tumor tissue, to elicit recognition and cytolytic inflammation from the CAR-T cells. Although survival was not assessed in this preliminary report, evidence of T cell infiltration and tumor regression was observed, marking a desperately needed achievement in the otherwise immunosuppressive microenvironment of DMG. These findings provide strong support for continued investigation into CAR-T cell therapy for patients with DMG. Currently, two other pediatric phase I trials are being conducted to assess the safety and efficacy of CAR-T cells directed against human epidermal growth factor receptor 2 (NCT03500991) and epidermal growth factor receptor variant III (NCT03638167), as these proteins have also been shown to be expressed at high levels in patients with DMG. Combination treatments consisting of multi-antigen targeting or cytokine overexpression CAR-T cells are also in development [42].

### Vaccines

Identification of H3 K27 alterations in at least 70% of patients with DIPG stimulated substantial interest in utilizing vaccines directed against histone alterations in these patients [120]. Vaccination against the most common H3K27 alteration, H3 K27M, induced mutation-specific CD4+ and CD8+ T cell responses in flank tumor models of DIPG, and a phase I clinical trial (NCT02960230) is ongoing to assess safety and efficacy of this vaccine in humans [121]. Recently, immunogenicity of H3K27M vaccination has been supported in patients with DIPG treated in combination with the toll-like receptor 3 (TLR3) agonist poly-ICLC and is being further investigated in a clinical trial (NCT02960230) [122]. Development of efficacious vaccines will require stimulation of a robust antitumor immune response and may require multi-antigen recognition, as brain tumors are known to adapt immune evasion mechanisms against single antigen-targeted treatments. Therefore, vaccination methods may be particularly efficacious when combined with immunotherapies that target multiple tumor subtypes and neoantigens to avoid selection of tumor escape variants.

### ICIs

ICIs inhibit proteins responsible for attenuating inflammatory responses of T cells. ICIs mostly consist of monoclonal antibodies directed against cytotoxic T lymphocyte-associated antigen-4 (CTLA-4), programmed cell death protein-1 (PD-1), or programmed cell death ligand protein-1 (PD-L1) [123]. ICIs have proven unsuccessful in providing benefit for patients with DMG, likely due to the near absence of TIL and the immunosuppressive nature of the DMG microenvironment [124]. In fact, a retrospective cohort study of patients with progressive DIPG demonstrated a nonsignificant survival benefit of patients treated with reirradiation or reirradiation + anti-PD-1 treatment with nivolumab [125]. However, ICIs may prove useful as adjuvant treatments with therapies that more broadly activate antitumor T cells, such as OVs (as described below), leading to expression of immune checkpoint proteins.

### Diffuse Pediatric-Type HGG, H3-Wildtype, and IDH-Wildtype

Diffuse pediatric-type HGG, H3-wildtype, and IDH-wildtype, are diagnosed with a combination of molecular and histological findings and, unlike adult brain tumor classification, they do not include the term “glioblastoma” as a clinical diagnosis. Historically, immunotherapies have not yielded improved outcomes in these patients, but recent preclinical and clinical studies have suggested a benefit for targeted immunotherapeutic approaches in patients with pHGG, H3-wildtype, and IDH-wildtype, indicating the first signs of clinical progress in decades [39].

### Oncolytic Viruses

OVs are viruses that selectively kill cancer cells and spare normal cells through native specificity or through genetic engineering approaches that negate infection in normal cells. OVs kill cancer cells through direct oncolysis via viral lytic cycles and/or through release of neoantigens and subsequent activation of an antitumor immune response. Unlike antigen- or receptor-specific immunotherapies (i.e., vaccines, ICIs, and CAR-T cells), OVs replicate in, and kill, a broad array of cancer cell types, including cancer stem cells. Therefore, OVs may be able to overcome intertumoral heterogeneity. We have extensively reviewed the various types of OVs in clinical trials for treatment of pHGG [126, 127]. Below, we summarize these findings and focus on the most widely researched OV, oncolytic herpes simplex virus-1 (oHSV). HSV is an enveloped, double-stranded DNA virus with a large genome (152 kb) that can be engineered to express immunostimulant genes, enabling a “trojan horse” type therapy that carries with it additional immunomodulator capability. The first FDA-approved OV was the oHSV talimogene laherparepvec (Tvec), which is engineered to express GM-CSF, and was approved to treat advanced melanoma [128]. Since the approval of Tvec in 2015, substantial interest has been given to the optimization of oHSV and combination treatments for brain tumors. The first completed clinical trial assessing oHSV safety and efficacy for children with pHGG was recently published and demonstrated safety in all 12 children (age 7–18 years) in the trial, as well as clinical and radiographic efficacy in 11 of 12 children [129]. Patients were administered up to 1 × 10^8^ plaque forming units (pfu) of the oHSV G207 with 5 Gy radiation to stimulate viral replication. Remarkably, no serious adverse events attributable to oHSV were reported, and no signs of viral shedding in the conjunctiva or saliva were observed, demonstrating the tumor specificity and systemic safety of oHSV. Most importantly, median overall survival was more than double historical controls (12.2 months vs. 5.6 months, 95% CI 8.0–16.4) and immunohistochemistry staining 2–9 months after administration of G207 revealed marked recruitment of tumor infiltrating CD4+ and CD8+ T cells in tissue that was previously void of these cells, suggesting G207 turned immunologically “cold” tumor tissue hot [84, 113]. Based on these findings, a multi-institutional phase II trial (NCT04482933) for children with recurrent HGG at relapse is opening with purpose of assessing progression-free survival. Clinical trials are also being conducted with oncolytic adenoviruses (AdV) with particular interest in alternate delivery routes of AdV using neural stem cell (NSC) or mesenchymal stem cell (MSC) delivery (NCT03330197) [130].

## Glioneuronal and Neuronal Tumors

Neuronal and glioneuronal tumors are a heterogeneous group of rare, mostly WHO grade I CNS neoplasms that arise as neuronal cells alone or as a mix of glial and neuronal cells [7, 26]. Despite their heterogeneity, neuronal and glioneuronal tumors are strongly associated with seizures in infants and children [131, 132]. Gangliogliomas, the most common type of glioneuronal tumor, are WHO grade I neoplasms appearing mostly in the temporal lobe with average diagnosis age of 11.4 years and predilection for males [133]. Desmoplastic neuroepithelial tumors (DNETs) are WHO grade I neoplasms favoring the cortex with incidence of 0.3 per million and average diagnosis age of 9 years [134]. Anaplastic gangliogliomas are very rare WHO grade III neoplasms that favor the temporal lobe with an incidence of only 0.02 per million [135]. The list of neuronal and glioneuronal tumors goes on and is currently evolving based on genetic alterations found in each tumor subtype [136]. Seizure management and maximum safe resection are the cornerstones for glioneuronal tumor treatment [137]. Prognosis is largely dependent on WHO classification of the given tumor subtype. While most low-grade glioneuronal tumors show excellent rates of recurrence-free survival on long-term follow-up, one retrospective study of anaplastic gangliogliomas (WHO grade III) revealed a recurrence rate at 5 years of 100% and median overall survival of only 24.7 months [131, 138, 139]. The rarity of many neuronal and glioneuronal subtypes creates a considerable challenge for developing future immunotherapies.

There is a paucity of literature surrounding the clinical use of immunotherapies treating neuronal and glioneuronal tumors. This is likely attributable to the excellent outcomes of low-grade tumor resection and rarity of many high-grade subtypes. There are two phase II and one phase I clinical trials testing immunotherapies that include patients with neuronal and glioneuronal tumors. The active phase II trial is studying the efficacy of anti-PD-1 mAb pembrolizumab to treat rare tumors that are metastatic or unresectable with primary outcome measures being tumor nonprogression rate and incidence of adverse events (NCT02721732). The other phase II trial is still recruiting to study combination immunotherapy using anti-CTLA4 mAb ipilimumab with anti-PD-1 mAb nivolumab in patients with advanced rare cancers with the primary outcome measure being overall tumor response rate (NCT02834013). A phase I trial was completed in September of 2015 demonstrating the safety of 10 mg/kg intravenous bevacizumab (NCT00876993), the anti-VEGF mAb with putative immunomodulatory effects in other cancer types [71, 72, 73, 140]. However, no phase II trials investigating bevacizumab in neuronal or glioneuronal tumors were found.

Beyond clinical trials, findings by Koelsche et al. [141] may give guidance on future investigations of immunotherapeutics. They observed frequent lymphocytic infiltrates within BRAFV600E-mutated gangliogliomas which suggests immunogenicity that may be exploited for developing future immunotherapies against BRAFV600E-mutated gangliogliomas. Analysis by Singh et al. [142] of DNA methylation data in glial and glioneuronal tumors revealed a positive correlation between monocyte proportion and expression of PD-L1 and PD-L2 in glioma/glioneuronal cells, suggesting monocyte-mediated immunogenicity in glioma/glioneuronal cells which may be an area of interest for developing future immunotherapies.

## Mesenchymal, Non-Meningothelial Tumors

The most common mesenchymal, non-meningothelial tumors of the CNS are hemangioblastomas [143]. These slow-growing, benign, WHO grade I vascular tumors are most commonly found in the cerebellum, brainstem, and spinal cord [143]. They commonly affect men between 30 and 50 years of age [144]. Common presenting symptoms generally arise due to local compression of neural structures, bleeding, and paraneoplastic complications [144]. First-line imaging is with Gadolinium-enhanced MRI. Von-Hippel Lindau disease accounts for nearly 20–30% of the patients who present with hemangioblastomas [145].

Treatment for this tumor depends upon the established diagnosis of VHL. Those without known VHL are treated with surgery rather than observation in order to help establish the diagnosis [146, 147]. Radiation therapy is considered in those with recurrent or residual disease [146, 147]. In patients with known VHL, treatment is individualized and involves a multidisciplinary team including neurosurgery, oncology, radiation oncology, and others [146]. Observation may be appropriate for asymptomatic tumors. Belzutifan, an HIF-2alpha inhibitor, has also shown promising results [148].

Literature on immunotherapy targeting hemangioblastomas is sparse. One study illustrating in vitro sensitization of lymphocytes to autologous RCC tumor cells in 2 VHL patients combined with an injection of IL-2 showed volume reduction of pulmonary metastasis without affecting the RCC volume [149].

## Pineal Tumors

Pineal tumors account for less than 1% of all primary brain tumors [150]. They most commonly affect males between ages 1 and 12 and have a predilection for those of Asian ancestry [150]. WHO classification delineates these as pineocytoma (WHO grade 1); pineal parenchymal tumor of intermediate differentiation (WHO grade 2 and 3); papillary tumor of the pineal region (WHO grade 2 and 3); desmoplastic myxoid tumor of the pineal region, SMARCB1-mutant and pineoblastoma (WHO grade 4) which is a highly malignant, infiltrative tumor with potential for leptomeningeal and extracranial dissemination and has a poor prognosis [7].

Pineoblastoma is most commonly seen in patients under 5 years of age. 90% of children present with hydrocephalus and 19% will have leptomeningeal dissemination. Median survival time in reported literature ranges from 16 to 25 months in the pediatric population [151, 152]. Immunotherapy for pineoblastoma remains off-label and investigational with sparse case reports following treatment with ICIs. These reports suggest multimodal immunotherapy may contribute to establishing disease control in pineoblastoma that has relapsed once remission is reached with standard oncologic care. A phase I clinical trial assessing the efficacy of the EGFR806-specific CAR-T cell for the treatment of several pediatric CNS tumors, including pineoblastoma, is currently in the recruitment phase (NCT03638167). A phase II clinical trial for the adult population using nivolumab (monoclonal antibody targeting PD-1) is currently underway (NCT03173950).

## Sellar and Pituitary Tumors

The 2021 WHO classification now follows the guidelines from the endocrine WHO classification and divides sellar tumors by their adenohypophyseal cell-lineage accounting for immunohistochemical hormone and transcription factor expression [7]. These include pituitary neuroendocrine tumors (PitNETs) − formerly known as pituitary adenomas − pituitary blastomas, adamantinomatous or papillary craniopharyngiomas, pituocytomas (groupsed together with granular cell tumor of the sellar region and spindle cell oncocytoma), and pituitary blastoma [7]. Other tumors that be present at the sellar region or adjacent to it include meningiomas and metastatic disease [153, 154].

Craniopharyngiomas are the most common sellar region tumor in the pediatric population. Immunotherapy for treatment of this tumor has traditionally centered on the use of interferon-alpha. Interferon-alpha can be placed intravenously or intracavitary to delay the need for surgery or radiation therapy [155]. This is a critical benefit for the developing brain against deleterious effects. It is hypothesized that this treatment works by promotion of cellular differentiation and inhibition of proliferation by modulating signaling mechanisms. Several important pathways that are implicated include phosphatidylinositol-3 kinase and Janus kinase/signal transducers and activators of transcription [155, 156].

Other pituitary tumors such as adenomas and malignant tumors in children are extremely rare, and data on immunotherapy exists mostly for the adult population. Two clinical trials in the adult population are underway using immunotherapy. A phase II clinical trial is currently assessing the effectiveness of nivolumab combined with ipilimumab in patients with aggressive pituitary tumors (NCT04042753). A second is a phase II clinical trial is investigating combination therapy with nivolumab and ipilimumab with its selection criteria includes patients with pituitary tumors (NCT02834013). Studies exploring immune-related biomarkers have proven promising [157]. Combination therapy with anti-CTLA-4 and anti-PD-1 monoclonal antibodies leads to infiltration with ICOS+ CD4+ effector T cells, a decreased in exhausted CD8+ T cells, and lead to expansion of activated CD8+ effector T cells [157]. Some promising candidates carried out by human protein-protein interaction include GAL, LMO4, STAT3, PD-L1, TGFB, and TGFBR3 [157].

## Spinal Tumors

Spinal cord gliomas are rare tumors, often with limited surgical options. These tumors are very infiltrative in nature limiting the ability to achieve significant surgical resection. While standard of care remains focused on maximal safe surgical resection followed by radiation therapy and chemotherapy, additional treatment strategies are urgently needed. While immunotherapy has yielded promising results in intracranial gliomas and astrocytomas, research on spinal cord gliomas is limited. The use of immunotherapy for the treatment of patients with spinal gliomas remains scarce due to considerable challenges with most of the landmark glioma immunotherapy trials targeting intracranial malignant glioma compared with spinal gliomas. Most of the literature at present is an extrapolation of intracranial targets with similar molecular makeup in the spinal cord.

Divergences between intracranial gliomas and spinal gliomas, for example, pose a significant challenge in the application of immunotherapy. Primary spinal cord tumors are exceedingly rare with an incidence of about 0.22 per 100,000 person-years [158]. Therefore, adequate enrollment in large clinical trials is challenging due to the rare nature of this disease. Additionally, the paucity of tumor specific antigens on spinal gliomas restricts the development of immunotherapies. It was assumed that spinal gliomas would have similar genetic mutations patterns as well as targetable antigens as their intracranial counterparts. However, recent advances in molecular studies have demonstrated that the molecular makeup is quite different in spinal gliomas [159]. Furthermore, small area of tumor volume in spinal gliomas along with their infiltrative nature make it exceedingly difficult to obtain tissue to work with to identify antigens.

Despite these challenges, the most promising target antigen for immunotherapy in the spine is H3.3K27M. This mutation is present in up to 40–50% of diffuse spinal cord astrocytic tumors. H3.3K27M has also been detected in pilocytic astrocytoma [159, 160]. Other potential target antigens include telomerase reverse transcriptase gene promoter (TERTp) mutation and the TP53 mutations which are present in up to 22.4% and 50% of spinal cord gliomas, respectively [159, 161].

H3.3K27M antigen has been targeted for T-cell receptor (TCR) T-cell therapy, CAR-T-cell therapy, and peptide vaccines, predominantly for intracranial lesions but also in the spine [118, 122, 162, 163]. Chheda et al. [163] demonstrated H3.3K37M specific T-cell response in intracranially xenografted midline glioma mice models which in turn led to a multicenter pilot study to evaluate safety, immunoreactivity, and efficacy of a vaccine. This vaccine used a 10 amino acid peptide spanning position 26–35 on the H3.3K27M protein in DMGs [122]. In this small pilot trial, 29 patients with DMGs received this peptide and were stratified based on location of the tumors. 19 patients with tumors in the pons were in stratum A and the other 10 were enrolled in stratum B (7 thalamic, 2 pontine-centered tumors with extension into cerebellum and cervical spine, and 1 spinal cord tumor in the thoracic spine). Outcomes were reported for the entire stratum, not for the individual patient with the spinal cord DMG. Nevertheless, 7 of the 29 patients showed evidence of response with a 25% postvaccination increase in H3.3K27M reactive CD8+ T cells. Patients in this group had a mean overall survival of 16.3 months postinjection when compared to the overall survival of 9.9 months in the nonresponder group. These results led to a multicenter phase I/II study that aims to evaluate the safety, immunoreactivity, and efficacy of combination therapy with the H3.3K27M26-35 peptide vaccine and nivolumab in patients with newly diagnosed DMGs, including spinal tumors (NCT02960230).

## Conclusion

The current landscape for immunotherapy in pediatric brain tumors is still in its early days, but results in certain tumors have been promising (Table 1). Since the introduction of ICIs, there has been an explosion of new targets and immunotherapy approaches for cancer treatment. In the age of targeted therapy, genetic tumor profiling, and many ongoing clinical trials, their use will likely become an increasingly effective tool in the neuro-oncologist's armamentarium.

## Conflict of Interest Statement

The authors have no conflicts of interest to declare.

## Statement of Ethics

An ethics statement is not applicable because this study is based exclusively on published literature. Written informed consent is not applicable because this study is based exclusively on published literature.

## Funding Sources

Research reported in this publication was supported in part by the National Institute of Neurological Disorders and Stroke of the National Institutes of Health (R25NS079188) to DEO. The content is solely the responsibility of the authors and does not necessarily represent the official views of the National Institutes of Health. This study was also completed, while DEO was a Cornwall Clinical Scholar supported by the University of Alabama at Birmingham.

## Author Contributions

DEO and SG: conception and design, literature review, manuscript draft and revision, and approval of final version; TJA, PM, JG, NMBL, EG, and RKD: literature review, manuscript draft and revision, and approval of final version; JMJ: conception and design, manuscript revision, and approval of final version.

## Data Availability Statement

All data generated or analyzed during this study are included in this article. Further inquiries can be directed to the corresponding author.

## Figures and Tables

**Table 1:** Ongoing and completed clinical trials of immunotherapy for the treatment of pediatric brain tumors

Tumor type	Tumor	Category	Study title	Phase	Immunotherapy agent	Immunotherapy type	Mechanism/target	Enrollment or estimated enrollment, *n*	Duration	NCT number	Status	Pubmed ID if published results
CPT	CPC	Recurrent	EGFR806-specific CART Cell Locoregional Immunotherapy for EGFR-positive Recurrent or Refractory Pediatric CNS Tumors	Phase I	EGFR806-specific CART cell	CAR-T	EGFR+ tumors	36	March 2019–March 2025	NCT03638167	Recruiting	
	
	CPC	Recurrent	Study of B7-H3-Specific CAR T Cell Locoregional Immunotherapy for Diffuse Intrinsic Pontine Glioma/Diffuse Midline Glioma and Recurrent or Refractory Pediatric Central Nervous System Tumors	Phase I	SCRI-CARB7H3(s); B7H3-specific CART cell	CAR-T	B7H3+ tumors	90	December 2019–May 2026	NCT04185038	Recruiting	
	
	CPC	Recurrent	HER2-specific CART Cell Locoregional Immunotherapy for HER2-positive Recurrent/ Refractory Pediatric CNS Tumors	Phase I	HER2-specific CART cell	CAR-T	HER2+ tumors	48	July 2018—July 2024	NCT03500991	Recruiting	

Embryonal tumors	MB	Recurrent	A Study to Evaluate the Safety and Efficacy of Nivolumab Monotherapy and Nivolumab in Combination With Ipilimumab in Pediatric Participants With High Grade Primary Central Nervous System (CNS) Malignancies	Phase II	Nivolumab and Ipilimumab	ICI	Antibodies to PD-1 and CTLA-4	166	June 2017–January 2022	NCT03130959	Completed	
	
	MB	Recurrent	Pembrolizumab in Treating Younger Patients With Recurrent, Progressive, or Refractory High-Grade Gliomas, Diffuse Intrinsic Pontine Gliomas, Hypermutated Brain Tumors, Ependymoma or Medulloblastoma	Phase I	Pembrolizumab (MK-3475)	ICI	Antibodies to PD-1	110	May 2015–December 2024	NCT02359565	Recruiting	
	
	MB	Recurrent	Durvalumab in Pediatric and Adolescent Patients	Phase I	Durvalumab (MEDI4736)	ICI	Antibodies to PD-1	36	July 2016–December 2020	NCT02793466	Unknown	
	
	MB	Recurrent	Expanded Natural Killer Cell Infusion in Treating Younger Patients With Recurrent/Refractory Brain Tumors	Phase I	Autologous ex vivo-expanded NK cells	NK cell therapy	Intraventricular activated NK cells	12	March 2015–August 2020	NCT02271711	Completed	
	
	MB	Recurrent	Study of B7-H3-Specific CAR T Cell Locoregional Immunotherapy for Diffuse Intrinsic Pontine Glioma/Diffuse Midline Glioma and Recurrent or Refractory Pediatric Central Nervous System Tumors	Phase I	CRI-CARB7H3(s); B7H3-specific CART cell	CAR-T	B7H3-specific CAR and EGFRt	90	December 2019-May 2026	NCT04185038	Recruiting	
	
	MB	Recurrent	EGFR806-specific CART Cell Locoregional Immunotherapy for EGFR-positive Recurrent or Refractory Pediatric CNS Tumors	Phase I	EGFR806-specific CART cell	CAR-T	EGFR806-specific CAR and EGFRt	36	March 2019–March 2025	NCT03638167	Recruiting	
	
	MB	Recurrent	HER2-spedfic CART Cell Locoregional Immunotherapy for HER2-positive Recurrent/ Refractory Pediatric CNS Tumors	Phase I	HER2-specific CART cell	CAR-T	HER2+ tumors	48	July 2018–July 2024	NCT03500991	Recruiting	
	
	MB	Recurrent	177Lu-DTPA-Omburtamab Radioimmunotherapy for Recurrent or Refractory Medulloblastoma	Phase 1/2177LU-DTPA-omburtamab	Radioimmunotherapy	B7H3+ tumors	40	September 2021–December 2024	NCT04167618	Recruiting	
	
	MB	Recurrent	Radiolabeled Monoclonal Antibody Therapy in Treating Patients With Refractory, Recurrent, or Advanced CNS or Leptomeningeal Cancer	Phase I	Iodine-131 MOAB 8H9	Radioimmunotherapy	Intrathecal radiolabeled 8H9 antibody	120	July 2004–July 2025	NCT00089245	Active, not recruiting	
	
	MB	Recurrent	Radiolabeled Monoclonal Antibody Therapy in Treating Patients With Primary or Metastatic Brain Cancers	Phase I	Iodine-131 monoclonal antibody 81C6	Radioimmunotherapy	Intraventricular radiolabeled 81C6 antibody	Unknown	February 1993–March 2010	NCT00002752	Completed	18287339
	
	MB	De Novo	Intrathecal Radioimmunotherapy, Radiation Therapy, and Chemotherapy After Surgery in Treating Patients With Medulloblastoma	Phase II	Iodine-131 monoclonal antibody 3F8	Radioimmunotherapy	Intrathecal radiolabeled 3F8 antibody	6	February 2003–June 2019	NCT00058370	Completed	28940863
	
	MB	Recurrent	PEP-CMV in Recurrent MEdulloblastoma/ Malignant Glioma	Phase I	PEP-CMV vaccine	Vaccine	Neurotropic CMV vaccine	30	June 2018–December 2024	NCT03299309	Recruiting	
	
MB	Recurrent	Clinical Trial to Assess the Safety and Efficacy of AloCELYVIR With Newly Diagnosed Diffuse Intrinsic Pontine Glioma (DIPG) in Combination With Radiotherapy or Medulloblastoma in Monotherapy (AloCELYVIR)	Phase I/IIAloCELYVIR	OV	Mesenchymal stem cells infected with an oncolytic Adenovirus, ICOVIR-5	12	April 2019–October 2024	NCT04758533	Recruiting	
	
MB	Recurrent	HSV G207 in Children With Recurrent or Refractory Cerebellar Brain Tumors	Phase I HSV G207	OV	Intratumoral G207 infusion	15	September 2019–September 2024	NCT03911388	Recruiting	
	
MB	Recurrent	Phase lb Study PVSRIPO for Recurrent Malignant Glioma in Children	Phase I Oncolytic polio/ rhinovirus recombinant (PVSRIPO)	OV	Intratumoral PVSRIPO	12	December 2017-March 2022	NCT03043391	Active, not recruiting	
	
MB	Recurrent	Modified Measles Virus (MV-NIS) for Children and Young Adults With Recurrent Medulloblastoma or Recurrent AT/RT	Phase I Modified measles virus (MV-NIS)	OV	Intratumoral or intrathecal infusion of MV-NIS	46	February 2017–May 2023	NCT02962167	Recruiting	
	
MB	Recurrent	Wild-Type Reovirus in Combination With Sargramostim in Treating Younger Patients With High-Grade Relapsed or Refractory Brain Tumors	Phase I Replication competent reovirus (Reolysin)	OV	Reolysin + GM-CSF therapy	6	June 2015–January 2025	NCT02444546	Active, not recruiting	
	
AT/RT	Recurrent	Modified Measles Virus (MV-NIS) for Children and Young Adults With Recurrent Medulloblastoma or Recurrent AT/RT	Phase I Modified measles virus (MV-NIS)	OV	Intratumoral or intrathecal infusion of MV-NIS	46	February 22, 2017-May 1, 2023	NCT02962167	Recruiting	
	
AT/RT	Recurrent	Tiragolumab and Atezolizumab for the Treatment of Relapsed or Refractory SMARCB1 or SMARCA4 Deficient Tumors	Phase II Tiragolumab and atezolizumab	ICI	Antibodies to TIGIT and PD-L1	78	June 16, 2022–July 31, 2025	NCT05286801	Not yet recruiting	
	
AT/RT	Recurrent	Study of B7-H3-Specific CAR T Cell Locoregional Immunotherapy for Diffuse Intrinsic Pontine Glioma/Diffuse Midline Glioma and Recurrent or Refractory Pediatric Central Nervous System Tumors	Phase I B7-H3-specific CAR T Cell	CAR-T	B7H3+ tumors	90	December 11, 2019-May 2041	NCT04185038	Recruiting	
	
AT/RT	Recurrent	EGFR806-specific CART Cell Locoregional Immunotherapy for EGFR-positive Recurrent or Refractory Pediatric CNS Tumors	Phase I EGFR806-specific CART Cell	CAR-T	EDFR + tumors	36	March 19, 2019-March 2040	NCT03638167	Recruiting	
	
AT/RT	Recurrent	HER2-specific CART Cell Locoregional Immunotherapy for HER2-positive Recurrent/ Refractory Pediatric CNS Tumors	Phase I HER2-specific CART cell	CAR-T	HER2+ tumors	48	July 26, 2018− July 26, 2039	NCT03500991	Recruiting	
	
AT/RT	Recurrent	Aflac ST0901 CHOANOME–Sirolimus in Solid Tumors (Aflac ST0901)	Phase I Sirolimus	ICI	mTOR inhibitor	18	April 2011− August 9, 201 7	NCT01331135	Completed	31876107
	
AT/RT	Recurrent	Wild-Type Reovirus in Combination With Sargramostim in Treating Younger Patients With High-Grade Relapsed or Refractory Brain Tumors	Phase I Wild-type reovirus	OV	Reovirus + Sargramostim	6	June 21, 2015–January 1, 2025	NCT02444546	Active, not recruiting	
	
AT/RT	De Novo	Tazemetostat + Nivo/lpi in INI1-Neg/SMARCA4-Def Tumors	Phase I/IINivolumab and ipilimumab	ICI	Antibodies to PD-1 and CTLA-4	49	September 2022–February 1, 2029	NCT05407441	Not yet recrutiing	
	
AT/RT	Recurrent	Ribociclib and Everolimus in Treating Children With Recurrent or Refractory Malignant Brain Tumors	Phase I Ribociclib	ICI	CDK 4/6 Inhibitor	22	January 13, 2018–April 1, 2020	NCT03387020	Completed	33547201
	
AT/RT	Recurrent	Lenalidomide in Treating Young Patients With Recurrent, Progressive, or Refractory CNS Tumors	Phase I Lenalidomide	Immunemodulator	T cell proliferation	45	November 2004-Unknown	NCT00100880	Completed	18056189
	
	AT/RT	De Novo	Nivolumab and Ipilimumab in Treating Patients With Rare Tumors	Phase II	Nivolumab and ipilimumab	ICI	Antibodies to PD-1 and CTLA-4	818	January 13, 2017–October 31, 2023	NCT02834013		
	
	AT/RT	Recurrent	Phase lb Study PVSRIPO for Recurrent Malignant Glioma in Children	Phase I	Poliovirus (PVSRIPO)	OV	Intracerebral delivery via convection-enhanced delivery (CED)	12	December 5, 2017–March 1, 2022	NCT03043391	Active, not recruiting	
	
	PNET	Recurrent	Vaccine Immunotherapy for Recurrent Medulloblastoma and Primitive Neuroectodermal Tumor (Re-MATCH)	Phase II	Autologous tumor-specific T cell immunotherapy (TTRNA-xALT) plus TTRNA-loaded DC vaccine	Vaccine	DC vaccine	25	September 7, 2010–December 30, 2022	NCT01326104	Active, not recruiting	
	
	PNET	Recurrent	Phase I/II: Decitabine/Vaccine Therapy in Relapsed/Refractory Pediatric High Grade Gliomas/Medulloblastomas/CNS PNETs	Phase I/II Autologoud DCs	Vaccine	DC vaccine	1	April 2015–July 2016	NCT02332889	Terminated	
	
	PNET	Recurrent	HSV G207 Alone or With a Single Radiation Dose in Children With Progressive or Recurrent Supratentorial Brain Tumors	Phase I	Oncolytic herpes simplex virus-1 (HSV) (G207)	OV	G207 HSV virus with/without radiation	12	May 2016–December 2022	NCT02457845	Active, not recruiting	33838625
	
	PNET	Recurrent	Iodine I 131 Monoclonal Antibody 3F8 in Treating Patients With Central Nervous System Cancer or Leptomeningeal Cancer	Phase II	Iodine I 131 monoclonal antibody 3F8	Radioimmunotherapy	Intrathecal radiolabeled 3F8 antibody	78	January 2006–January 2023	NCT00445965	Active, not recruiting	
	
	PNET	Recurrent	Phase lb Study PVSRIPO for Recurrent Malignant Glioma in Children	Phase I	Poliovirus (PVSRIPO)	OV	Intracerebral delivery via convection-enhanced delivery (CED)	12	December 5, 2017–March 1, 2022	NCT03043391	Active, not recruiting	
	
	PNET	Recurrent	Durvalumab in Pediatric and Adolescent Patients	Phase I	Durvalumab	ICI	PD-1 inhibitor	36	July 2016–December 2020	NCT02793466	Unknown	
	
	PNET	Recurrent	EGFR806-specific CART Cell Locoregional Immunotherapy for EGFR-positive Recurrent or Refractory Pediatric CNS Tumors	Phase I	EGFR806-specific CART Cells	CAR-T	EGFR+ tumors	36	March 19, 2019-March 2040	NCT03638167	Recruiting	
	
	PNET	Recurrent	HER2-specific CART Cell Locoregional Immunotherapy for HER2-positive Recurrent/ Refractory Pediatric CNS Tumors	Phase I	HER2-specific CART Cells	CAR-T	HER2+ tumors	48	July 26, 2018–July 26, 2039	NCT03500991	Recruiting	
	
	PNET	Recurrent	Wild-Type Reovirus in Combination With Sargramostim in Treating Younger Patients With High-Grade Relapsed or Refractory Brain Tumors	Phase I	Wild-type reovirus	OV	Reovirus + Sargramostim	6	June 21, 2015–January 1, 2025	NCT02444546	Active, not recruiting	
	
	PNET	Recurrent	Chemotherapy and Vaccine Therapy Followed by Bone Marrow or Peripheral Stem Cell Transplantation and Interleukin-2 in Treating Patients With Recurrent or Refractory Brain Cancer	Phase II	Autologous tumor cell vaccine, sargramostim (GM-CSF), and IL2	Vaccine	GM-CSF and IL2	Unknown	August 1998–October 2004	NCT00014573	Completed	
	
	PNET	Recurrent	Vaccine Therapy and Interleukin-2 in Treating Young Patients With Relapsed or Refractory Ewing's Sarcoma or Neuroblastoma	Phase I	Epstein-Barr virus-transformed B-lymphoblastoid cells and IL2	Vaccine	CTL, IL2	10	November 2004-Unknown	NCT00101309	Unknown	
	
	PNET	Recurrent	Study of B7-H3-Specific CAR T Cell Locoregional Immunotherapy for Diffuse Intrinsic Pontine Glioma/Diffuse Midline Glioma and Recurrent or Refractory Pediatric Central Nervous System Tumors	Phase I	B7-H3-specific CAR T cell	CAR-T	B7H3+ tumors	90	December 11, 2019–May 2041	NCT04185038	Recruiting	
	
	PNET	Recurrent	Lenalidomide in Treating Young Patients With Recurrent, Progressive, or Refractory CNS Tumors	Phase I	Lenalidomide	Immunemodulator	T cell proliferation	45	November 2004-Unknown	NCT00100880	Completed	18056189
	
	PNET	Recurrent	Radiolabeled Monoclonal Antibody Therapy in Treating Patients With Refractory, Recurrent, or Advanced CNS or Leptomeningeal Cancer	Phase I	Iodine-131 MOAB 8H9	Radioimmunotherapy	Intrathecal radiolabeled 8H9 antibody	120	July 2004–July 2025	NCT00089245	Active, not recruiting	
	
	PNET	Recurrent	Chemo-immunotherapy Using Ibrutinib Plus Indoximod for Patients With Pediatric Brain Cancer	Phase I	Ibrutinib, indoximod	ICI	BTK inhibitor, IDO inhibitor	37	February 8, 2022–April 2025	NCT05106296		

Cranial nerve sheath tumors	Nerve sheath tumors	De Novo	Neoadjuvant Nivolumab Plus Ipilimumab for Newly Diagnosed Malignant Peripheral Nerve Sheath Tumor	Phase I	Nivolumab and ipilimumab	ICI	Antibodies to PD-1 and CTLA-4	18	June 2021–June 2025	NCT04465643	Recruiting	
	
	Nerve sheath tumors	Recurrent	Bevacizumab and Temsirolimus Alone or in Combination With Valproic Acid or Cetuximab in Treating Patients With Advanced or Metastatic Malignancy or Other Benign Disease	Phase I	Bevacizumab	anti-VEGF	Antibodies to VEGF	155	March 2012–March 2022	NCT01552434	Active	
	
	Nerve sheath tumors	Recurrent	B7H3 CAR T Cell Immunotherapy for Recurrent/ Refractory Solid Tumors in Children and Young Adults	Phase I	Second generation 4-1 BBζ B7H3-EGFRt-DHFR(selected) and second generation 4-1 BBζ CD19–Her2tG	CAR-T	B7H3+ tumors	68	July 2020–December 2025	NCT04483778	Recruiting	
	
	Nerve sheath tumors	Recurrent	EGFR806 CART Cell Immunotherapy for Recurrent/Refractory Solid Tumors in Children and Young Adults	Phase I	Second generation 4-1 BBζ EGFR806-EGFRt and second generation 4-1 BBC CD19-Her2tG	CAR-T	EGFR+ tumors	36	June 2019–June 2023	NCT03618381	Recruiting	
	
	Nerve sheath tumors	Recurrent	B7-H3-Specific Chimeric Antigen Receptor Autologous T-Cell Therapy for Pediatric Patients With Solid Tumors (3CAR)	Phase I	B7H3-specific CART cells	CAR-T	B7H3+ tumors	32	May 2022-March 2026	NCT04897321	Not yet recruiting	

Ependymal tumors	Ependymoma	Recurrent	Immunotherapy for Recurrent Ependymomas in Children Using Tumor Antigen Peptides With Imiquimod	Phase I	Vaccination with HLA-A2 restricted peptides, combined with imiquimod	Vaccine	IL-13Rα2, EphA2, Survivin	24	August 2012-December 2023	NCT01795313	Recruiting	
	
	Ependymoma	Recurrent	HER2-specific Chimeric Antigen Receptor (CAR) T Cells for Children With Ependymoma	Phase I	HER2-specific CART cells	CAR-T	HER2	50	May 2022-July 2025	NCT04903080	Recruiting	
	
	Ependymoma	Recurrent and incomplete resection	Study of the Effect of GM-CSF on Macrophages in Ependymoma	Phase I	Granulocyte macrophage colony stimulation factor	Glycoprotein	Supports survival, clonal expansion, and differentiation of hematopoietic progenitor cells	6	June 2013-December 2022	NCT04408092	Active	
	
	Ependymoma	Recurrent	A Phase I Study of AdV-tk + Prodrug Therapy in Combination With Radiation Therapy for Pediatric Brain Tumors	Phase I	AdV-tk	Gene-mediated cytotoxic immunotherapy	Leads to local creation of nucleotide analogs that result in the death of dividing cancer cells and the consequent release of tumor neoantigens	8	October 2010–June 2021	NCT00634231	Completed	30883662
	
	Ependymoma	Recurrent	Expanded Natural Killer Cell Infusion in Treating Younger Patients With Recurrent/Refractory Brain Tumors	Phase I	Autologous NK cells	Autologus expanded NK cells	NK cells release cytotoxic granules containing perforin and granzymes to directly lyse tumor cells	12	March 2015-August 2020	NCT02271711	Completed	32152626
	
Tumor type	Tumor	Category	Study title	Phase Immunotherapy agent	Immunotherapy type	Mechanism/target	Enrollment or estimated enrollment, *n*	Duration	NCT number	Status	Pubmed ID if published results
	
	Ependymoma	Recurrent	HER2-Specific CART Cell TherapyHER2-specific CART Cell Locoregional Immunotherapy for HER2-positive Recurrent/Refractory Pediatric CNS Tumors	Phase I HER2-specific CART cells	CAR-T	HER2	48	July 2018-July 2024	NCT03500991	Recruiting	
	
	Ependymoma	Recurrent	Everolimus for Children with Recurrent or Progressive Ependymoma	Phase II Everolimus	Immune checkpoint inhibitor	mTOR	11	February 2015–Juiy 2022	NCT02155920	Active	

Germ cell tumors	Several	Recurrent	HER2-Specific CART Cell TherapyHER2-specific CART Cell Locoregional Immunotherapy for HER2-positive Recurrent/Refractory Pediatric CNS Tumors	Phase I HER2-specific CART cells	CART	HER2	48	July 2018-July 2024	NCT03500991	Recruiting	
	
	Several	Recurrent	Imatinib Mesylate in Treating Patients With Progressive, Refractory, or Recurrent Stage II or Stage III Testicular or Ovarian Cancer	Phase II Imatinib mesylate	Small molecule	c-KIT	32	June 2002-October 2003	NCT00042952	Terminated	16462496

Gliomas	Pediatric LGG	Recurrent	A Trial of Poly-ICLC in the Management of Recurrent Pediatric Low Grade Gliomas (Poly-ICLC)	Phase I/IIPoiy-ICLC	Stabilized double-stranded RNA	TLR-3 and RIG-I	50	November 2016-April 2022	NCT02960230	Completed	PMC6012063
	
	Pediatric LGG	Recurrent and primary	Vinblastine +/− Bevacizumab in Children With Unresectable or Progressive Low Grade Glioma	Phase II Bevacizumab	Antibody	VEGF	150	July 2016-March 2020	NCT02840409	Recruiting	
	
	Optic gliomas	Recurrent or refractory	A Phase II Study Of Pegylated Interferon ALFA-2b in Children With Recurrent or Refractory and Radiographically or Clinically Progressive Juvenile Pilocytic Astrocytomas and Optic Pathway Gliomas	Phase II Peginterferon alpha-2b	IFN	Binds and activates type 1 human interferons causing activation of JAK/STAT pathway	9	January 2015-January 2022	NCT02343224	Completed	PMC9165184
	
	Juvenile pylocytic astrocytomas	Recurrent or refractory	A Phase II Study Of Pegylated Interferon ALFA-2b in Children With Recurrent or Refractory and Radiographically or Clinically Progressive Juvenile Pilocytic Astrocytomas and Optic Pathway Gliomas	Phase II Peginterferon alpha-2b	IFN	Binds and activates type 1 human interferons causing activation of JAK/STAT pathway	9	January 2015-January 2022	NCT02343224	Completed	PMC9165184
	
	H3K27M DIPG or spinal DMG	Recurrent	Phase I Clinical Trial of Autologous GD2 Chimeric Antigen Receptor (CAR) T Cells (GD2CART) for Diffuse Intrinsic Pontine Gliomas (DIPG) and Spinal Diffuse Midline Glioma (DMG)	Phase I GD2 CART cells	CART	GD2	54	June 2020–July 2027	NCT04196413	Recruiting	35130560
	
	H3K27M DIPG, DMG, or spinal DMG	Primary	H3.3K27M Specific Peptide Vaccine Combined With Poly-ICLC With and Without PD-1 Inhibition Using Nivolumab for the Treatment of Newly Diagnosed HLA-A2 (02:01)+ H3.3K27M Positive Diffuse Intrinsic Pontine Glioma (DIPG) and Newly Diagnosed HLA-A2 (02:01)+ H3.3K27M Positive Gliomas	Phase I/IINivolumab (PD-1 inhibitor)	Vaccine + ICI	H3.3K27M peptide vaccine + PD-1 inhibition	50	November 2016–November 2024	NCT02960230	Active, Not Recruiting	
	
	Diffuse high-grade glioma	Recurrent	Phase I Clinical Trial of HSV G207 Alone or With a Single Radiation Dose in Children With Recurrent Supratentorial Brain Tumors	Phase I oHSV (G207)	Oncolytic virus	Herpes simplex virus-1 engineered to kill tumor Cells	12	May 2016–December 2022	NCT02457845	Active, Not Recruiting	33838625
	
	Diffuse high-grade glioma	Recurrent	Phase II Clinical Trial of HSV G207 With a Single 5 Gy Radiation Dose in Children With Recurrent High-Grade Glioma	Phase II oHSV (G207)	Oncolytic virus	Herpes simplex virus-1 engineered to kill tumor cells	30	December 2022–December 2026	NCT04482933	Not Yet Recruiting	
	
	DIPG	Recurrent	A Phase I/II Study of Ad-RTS-hlL-12, an Inducible Adenoviral Vector Engineered to Express hlL-12 in the Presence of the Activator Ligand Veledimex in Pediatric Brain Tumor Subjects	Phase I/IIAd-RTS-hIL-12	OV	Inducible adenoviral vector engineered to express hlL-12 in the presence of the activator ligand veledimex	6	November 2017-November 2021	NCT03330197	Terminated	PMC6216124
	
Tumor type	Tumor	Category	Study title	Phase	Immunotherapy agent	Immunotherapy type	Mechanism/target	Enrollment or estimated enrollment, *n*	Duration	NCT number	Status	Pubmed ID if published results

Glioneuronal and neuronal tumors	Ganglioglioma	Recurrent	Study of Irinotecan and Bevacizumab With Temozolomide in Refractory/Relapsed Central Nervous System (CNS) Tumors	Phase I	Bevacizumab	Radioimmunotherapy	Antibodies to VEGF	26	September 2008–September 2015	NCT00876993	Completed	35260913

Pineal tumors	Pineoblastoma	Recurrent	EGFR806-specific CART Cell Locoregional Immunotherapy for EGFR-positive Recurrent or Refractory Pediatric CNS Tumors	Phase I	EGFR806-specific CART cell	CAR-T	Autologous CD4+ and CD8+ T cells lentivirally transduced to express an EGFR806-specific CAR and EGFRt given via indwelling CNS catheter	36	March 2019–March 2025	NCT03638167	Recruiting	
	
	Pineoblastoma	Recurrent	Study of B7-H3-Specific CAR T Cell Locoregional Immunotherapy for Diffuse Intrinsic Pontine Glioma/Diffuse Midline Glioma and Recurrent or Refractory Pediatric Central Nervous System Tumors	Phase I	SCRI-CARB7H3 (s); B7H3-specific CART cell	CAR-T	Autologous CD4+ and CD8+ T cells lentivirally transduced to express a B7H3 specific CAR and EGFRt given via indwelling CNS catheter	90	December 2019-May 2026	NCT04185038	Recruiting	
	
	Pineoblastoma	Recurrent	HER2-specific CART Cell Locoregional Immunotherapy for HER2-positive Recurrent/ Refractory Pediatric CNS Tumors	Phase I	HER2-specific CART cell	CAR-T	Autologous CD4 and CD8 T cells lentivirally transduced to express a HER2 specific CAR and EGFRt given via indwelling CNS catheter	48	July 2018–July 2024	NCT03500991	Recruiting	

Sellar region tumors	Craniopharyngioma	Recurrent	DX-8951 f in Treating Children With Advanced Solid Tumors or Lymphomas	Phase I	Filgastrim with exatecan mesylate	G-CSF	Glycoprotein to stimulate bone marrow	N/A	September 1999–April 2004	NCT00004212	Completed	
	
	Craniopharyngioma	Recurrent	Tocilizumab in Children With ACP	Phase I	Tocilizumab	Antibody	Antibody blocking IL-6	27	June 2019–December 2024	NCT03970226	Recruiting	
	
	Craniopharyngioma	Recurrent	Peginterferon Alfa-2b in Younger Patients With Craniopharyngioma That is Recurrent or Cannot Be Removed By Surgery	Phase II	Peginterferon a1pha-2b	IFN	Binds and activates type 1 human interferons causing activation of JAK/STAT pathway	19	April 2014–December 2018	NCT01964300	Terminated	

Spine peds tumors	H3.K27MPrimary DMGs	H3.3K27M Peptide Vaccine With Nivolumab for Children With Newly Diagnosed DIPG and Other Gliomas	Phase I/IIK27M peptide + nivolumab	Vaccine + ICI	H3.3K27M peptide vaccine + PD-1 inhibition	50	November 2016–November 2024	NCT02960230	Active, Not Recruiting	

ICI, immune checkpoint inhibitors; N/A, not applicable; CAR-T, chimeric antigen receptort cells; IFN, interferon.
